# Effect of GS-441524 in combination with the 3C-like protease inhibitor GC376 on the treatment of naturally transmitted feline infectious peritonitis

**DOI:** 10.3389/fvets.2022.1002488

**Published:** 2022-10-28

**Authors:** Jinbao Lv, Yang Bai, Yingyun Wang, Liu Yang, Yipeng Jin, Jun Dong

**Affiliations:** College of Veterinary Medicine, China Agricultural University, Beijing, China

**Keywords:** feline infectious peritonitis, GS-441524, GC376, combination, field experiment

## Abstract

**Objectives:**

The main objectives of this study were to investigate the efficacy of the nucleotide analog GS-441524 in combination with the 3C-like protease inhibitor GC376 for the treatment of naturally aquired feline infectious peritonitis (FIP) and to test whether their combination shortens the dosing period and improves the cure rate.

**Methods:**

In total, 46 FIP-affected cats were enrolled in this experiment, including 36 with wet FIP (29 with abdominal effusion, six with thoracic effusion, and one with thoracic+abdominal effusion), and 10 with dry FIP. The cats were aged from 3 to 96 months. Thoracic+abdominal effusion, lymph-node puncture fluid and perirenal puncture fluid was collected from the affected cats for qPCR testing, and all 46 cats were positive for feline coronavirus (FCoV). The cats divided into different dose groups, all treated for 4 weeks: group 1 (GS-441524, 5 mg/kg.sc.q.24 h; GC376, 20 mg/kg.sc.q.12 h), group 2 (GS-441524, 2.5 mg/kg.sc.q.24 h; GC376, 20 mg/kg.sc.q.12 h), group 3 (GS-441524, 2.5 mg/kg.sc.q.24 h; GC376, 10 mg/kg.sc.q.12 h), and group 4 (GS-441524, 5 mg/kg.sc.q.24 h; GC376, 10 mg/kg.sc.q.12 h).

**Results:**

After the 4-week combination treatment, 45 of the 46 (97.8%) cats survived, and 43 of those became clinically normal. Two cats required longer (7 to 12 weeks) treatment to achieve full recovery. As of writing (10 months after completion of the trial), all 45 cats were alive and no relapse was observed.

**Conclusions and relevance:**

GS-441524 combined with GC376 can be safely and effectively used to treat FIP and reduces the treatment period to 4 weeks, with an excellent cure rate.

## Introduction

Feline infectious peritonitis (FIP) is an infectious and highly lethal systemic disease caused by feline coronavirus (FCoV) (1), which belongs to the *Alphacoronavirus* genus. Although FCoV and the new human coronavirus SARS CoV-2 (causing COVID-19) belong to different genera: *Alphacoronavirus* and *Betacoronavirus*, respectively, they share some virological and epidemiological features (2). Therefore, treatments for FCoV may have some relevance for treatment of severe acute respiratory syndrome coronavirus 2 (SARS CoV-2). FIP is one of the four major causes of feline mortality (3). In the past, the lack of an effective treatment meant that FIP was almost invariably fatal, and the clinical mortality rate as high as 90% (4). Affected cats present with two main types of disease, wet and dry, and the wet type is more frequently observed, accounting for 60–70% of cases of FIP (5). Wet-type disease is clinically characterized by the presence of thoracic effusion, abdominal effusion, or thoracic+abdominal effusion, peritonitis, and pleurisy, with characteristic pathological lesions clinical signs, such as granuloma-like changes in the thoracoabdominal cavity. Some affected cats also show ocular and neurological signs (6). In recent years, the nucleotide analog GS-441524 and the 3C-like protease inhibitor GC376 have been shown to have good efficacy for treatment of both laboratory-induced and naturally aquired FIP (7–9).

The 3C-like protease inhibitor (3CLpro) binds to the active site of the viral 3C protease, blocking its catalytic activity, and thus inhibits coronavirus replication *in vivo* (10). In a study of feline FCoV infection, Kim et al. demonstrated that GC376 effectively inhibits coronavirus replication in naturally infected cats (11). Pedersen et al. further showed that GC376 is safe and effective for the treatment of cats with various forms of natural clinical FCoV infection when the drug is used for 12–17 weeks (7).

GS-441524 is a small-molecule competitive inhibitor of nucleoside triphosphates (NTPs), developed from the precursor drug GS-5734, and GS-5734 can effectively prevent experimental Ebola virus in rhesus monkeys and inhibit epidemic zoonotic coronavirus (Middle East Respiratory Syndrome) in mouse infection model (12). GS-441524 is required to be phosphorylated intracellularly by cell kinases to nucleoside monophosphate, which is then phosphorylated to the active triphosphate metabolite (NTP). Active NTP analog acts as a competitor to natural nucleoside triphosphate in viral RNA synthesis (9), When a GS-441524 molecule is inserted into a transcription product, transcription is terminated prematurely, inhibiting the viral RNA transcription process. Therefore, GS-441524 has presumed antiviral activity against many RNA viruses. Pedersen et al. showed the efficacy of GS-441524 given for 12 weeks in the treatment of 32 cats naturally infected with FCoV (8). Another study showed that GS-441524 can be used to treat FIP with neurological presentation with similar efficacy. The optimal dose range for GS-441524 in cats with neurological FIP (5–10 mg/kg) was determined by Peter et al. in four cats (13).

Based on the findings discussed above, both GC376 and GS-441524 are efficacious in the treatment of FIP, although the two drugs have completely different mechanisms of inhibiting viral replication (10, 14). However, treatment with either GC376 or GS-441524 as stand-alone drugs, required long dosing periods was long (12 to 17 weeks), which is challenging for both pets and pet owners.

Therefore, the main objectives of this study were to investigate the efficacy of the nucleotide analog GS-441524 in combination with the 3C-like protease inhibitor GC376 in the treatment of naturally acquired FIP and to test whether the combination of GC376 and GS-441524 shortens the treatment period and improves the cure rate.

## Materials and methods

### Experimental animals

The experiment was conducted at the Animal Hospital of China Agricultural University (Beijing China). The experiment was conducted in random groups, and the cats who came to the hospital in the corresponding stage of the experiment cycle were randomly assigned to the corresponding dose group. Before the experiment, we fully communicated with the owners of the affected animals, describing the mechanism of action of the two drugs in detail, and all owners signed the Experimental Informed Letter. In total, 46 cats with suspected FIP, aged 3–96 months, abdominal effusion and/or thoracic effusion samples from wet cases, and lymph-node puncture fluid and perirenal puncture fluid from dry cases were used for quantitative PCR (qPCR), and all cats tested positive for FCoV by qPCR (15), including 29 with abdominal effusion, six with thoracic effusion, one with thoracic+abdominal effusion, and 10 with dry-type disease. The basic information on the 46 cats in the group is shown in Table 1.

**Table 1:** Basic information on enrolled cats.

**Group**	**Group serial number**	**Affected cat breeds**	**Gender**	**Age**	**Date of diagnosis**	**Type of disease**
Group 1 (CF1)	CF1-1	British short	M	1Y	2019/5/12	Dry (MLE, K)
	CF1-2	British short	M	3M	2019/5/16	Abdominal effusion (MLE)
	CF1-3	British short	F	1.5Y	2019/5/16	Thoracic effusion
	CF1-4	Muppet	MC	1Y	2019/5/18	Thoracic effusion (MLE)
	CF1-5	American Short	F	1Y	2019/5/16	Abdominal effusion
	CF1-6	British short blue cat	F	3M	2019/5/18	Abdominal effusion
	CF1-7	Shorthair cat	F	6M	2019/5/18	Abdominal effusion
	CF1-8	Tiger Spot String	M	8M	2019/5/18	Abdominal effusion (MLE)
	CF1-9	British short	M	4M	2019/5/18	Abdominal effusion
	CF1-10	Ear folded	M	1.5Y	2019/5/18	Abdominal effusion
	CF1-11	Garfield	M	1Y	2019/5/18	Abdominal effusion (MLE)
Group 2 (CF2)	CF2-1	Shorthair cat	FS	8Y	2019/5/25	Abdominal effusion
	CF2-2	British short	M	7Y	2019/5/25	Abdominal effusion (MLE)
	CF2-3	Mumbai Cat	MC	2Y	2019/5/25	Dry (K)
	CF2-4	British Short and Gold Gradient	M	8M	2019/5/25	Abdominal effusion (MLE)
	CF2-5	Muppet	MC	1Y	2019/5/26	Dry (MLE, K)
	CF2-6	Leopard Cat	M	1Y	2019/5/25	Dry (K)
	CF2-7	Shorthair cat	F	4M	2019/6/3	Abdominal effusion
	CF2-8	British short	F	3Y	2019/6/3	Abdominal effusion (MLE)
	CF2-9	American Short	M	1Y	2019/6/6	Abdominal effusion
	CF2-10	American Short	MC	2Y	2019/6/8	Thoracic+Abdominal effusion
	CF2-11	British short	F	4M	2019/6/8	Dry (K)
Group 3 (CF3)	CF3-1	Idyllic Cat	M	1.5Y	2019/9/19	Abdominal effusion
	CF3-2	Abyssinia	M	1Y	2019/9/19	Abdominal effusion (MLE)
	CF3-3	Idyllic Cat	MC	7Y	2019/9/21	Thoracic effusion
	CF3-4	Idyllic Cat	F	Not available	2019/9/21	Abdominal effusion
	CF3-5	British short	F	11M	2019/9/26	Abdominal effusion (MLE)
	CF3-6	American Short	MC	4Y	2019/9/26	Abdominal effusion
	CF3-7	British short	M	1Y	2019/9/26	Abdominal effusion (MLE)
	CF3-8	British Short Silver Gradient	FS	2Y	2019/9/26	Abdominal effusion
	CF3-9	Shorthair cat	MC	2Y	2019/9/26	Thoracic effusion
	CF3-10	Shorthair cat	M	4M	2019/9/26	Abdominal effusion (MLE)
	CF3-11	British short	M	1Y	2019/10/4	Abdominal effusion
	CF3-12	American Short	MC	4Y	2019/10/4	Abdominal effusion
Group 4 (CF4)	CF4-1	Garfield	MC	2Y	2019/10/4	Dry (MLE)
	CF4-2	British short	M	3M	2019/10/7	Abdominal effusion
	CF4-3	British short	F	5M	2019/10/10	Abdominal effusion
	CF4-4	Shorthair cat	F	6M	2019/10/17	Dry (K)
	CF4-5	Idyllic Cat	M	5M	2019/10/19	Thoracic effusion (MLE)
	CF4-6	British short	M	8M	2019/10/19	Abdominal effusion
	CF4-7	British short	M	5M	2019/10/24	Dry (MLE)
	CF4-8	Siam	MC	5Y	2019/10/19	Dry (MLE, K)
	CF4-9	Long-haired cats	M	3M	2019/10/31	Abdominal effusion
	CF4-10	Long-haired cats	F	5M	2019/11/2	Abdominal effusion
	CF4-11	Muppet	F	3Y	2019/11/9	Thoracic effusion
	CF4-12	Shorthair cat	F	6M	2019/11/9	Dry (MLE, K)

FS, female spayed; F, entire female; MC, male castrated; M, entire male; MLE, Mesenteric lymph node enlargement; K, kidney.

### Examinations

Clinical assessment of the cats included a physical examination (body temperature, body weight, mental status, appetite, level of dehydration, defecation and urination), routine hematology, serum biochemistry and an ultrasound examination, before enrollment. During the test period, all cats come to the hospital for examination every week, the main monitoring indices were body temperature, body weight, hematocrit (HCT), hemoglobin (HGB), white blood cell (WBC), total protein (TP), globulin (GLOB), albumin (ALB), the albumin–globulin ratio (A:G) and total bilirubin (TBIL). During the treatment period, the above indicators were monitored and recorded every week, and the average value within the group was calculated for each data, and the comparison between the groups was carried out.

### Drugs

GC376 was provided by WuXi AppTec (Wuxi, Jiangsu Province, China). It was prepared at a concentration of 50 mg/mL in a mixture of 10% anhydrous ethanol and 90% polyethylene glycol 400 (PEG 400) (7), and the recommended dose was 10–20 mg/kg given subcutaneously (sc) every 12 h (q12 h) (0.2–0.4 mL/kg).

GS-441524 was provided by Bellen Chemistry Co., Ltd. It was dissolved to a concentration of 12.5 mg/mL in 5% ethanol, 30% propylene glycol, 45% PEG 400, and 20% water, and adjusted to pH 3–4 with concentrated HCl (8). The recommended dose was 2.5–5.0 mg/kg.sc.q.24 h.

### Treatment options

Cats were assigned into one of the four different treatment groups (Table 2). The test regimen was combined dosing for 4 weeks.

**Table 2:** The treatment regiment.

**Group**	**GS441524**	**GC376**
CF1	5 mg/kg.sc.q.24 h	GC376, 20 mg/kg.sc.q.12 h
CF2	2.5 mg/kg.sc.q.24 h	GC376, 20 mg/kg.sc.q.12 h
CF3	2.5 mg/kg.sc.q.24 h	GC376, 10 mg/kg.sc.q.12 h
CF4	5 mg/kg.sc.q.24 h	GC376, 10 mg/kg.sc.q.12 h

sc.q.24 h, Subcutaneous injection every 24 h; sc.q.12h, Subcutaneous injection every 12 h.

## Results

### Signalment

**Figure 1:**
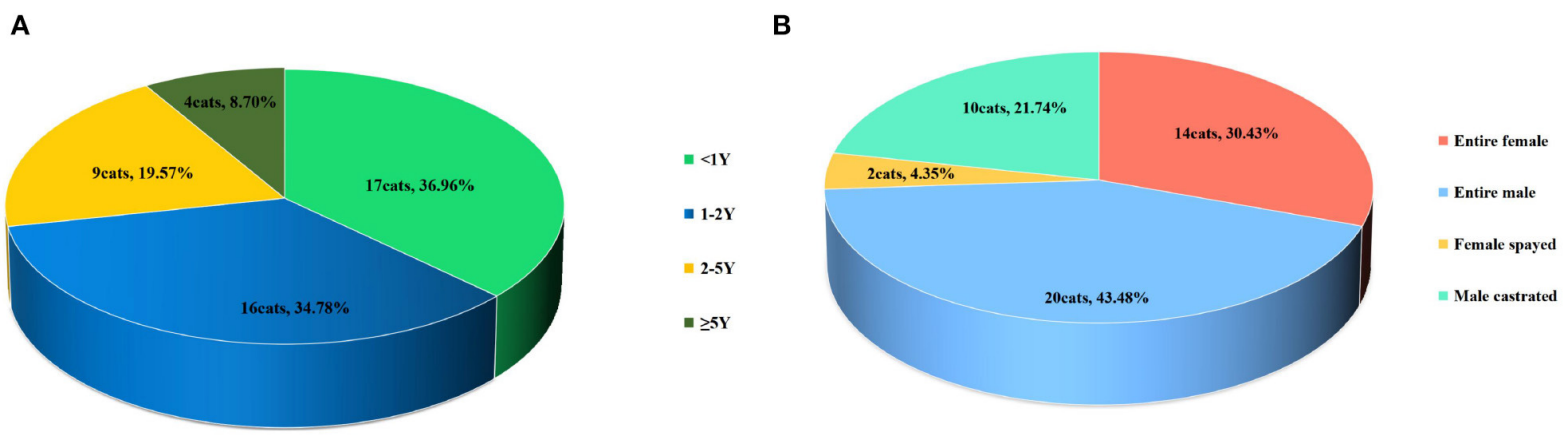
**(A)** Age proportion of the cats. **(B)** Sex proportion of the cats.

The age, sex, and disease type of the enrolled cats are shown in Table 1. The age distribution of the affected cats is shown in Figure 1A: 34.78% of cats were < 1 year old, 36.96% of cats were 1–2 years old, 19.57% of cats were 2–5 years old, and 8.70% of cats were ≥ 5 years old. The overall age of the affected cats was low, but cats of all ages had the disease.

The sex distribution of the affected cats is shown in Figure 1. The total proportion of entire male and male castrated cats was 66%, and the total proportion of entire female and female spayed cats was 34%. Thus, the proportion of male cats affected was about twice that of female cats.

**Figure 2:**
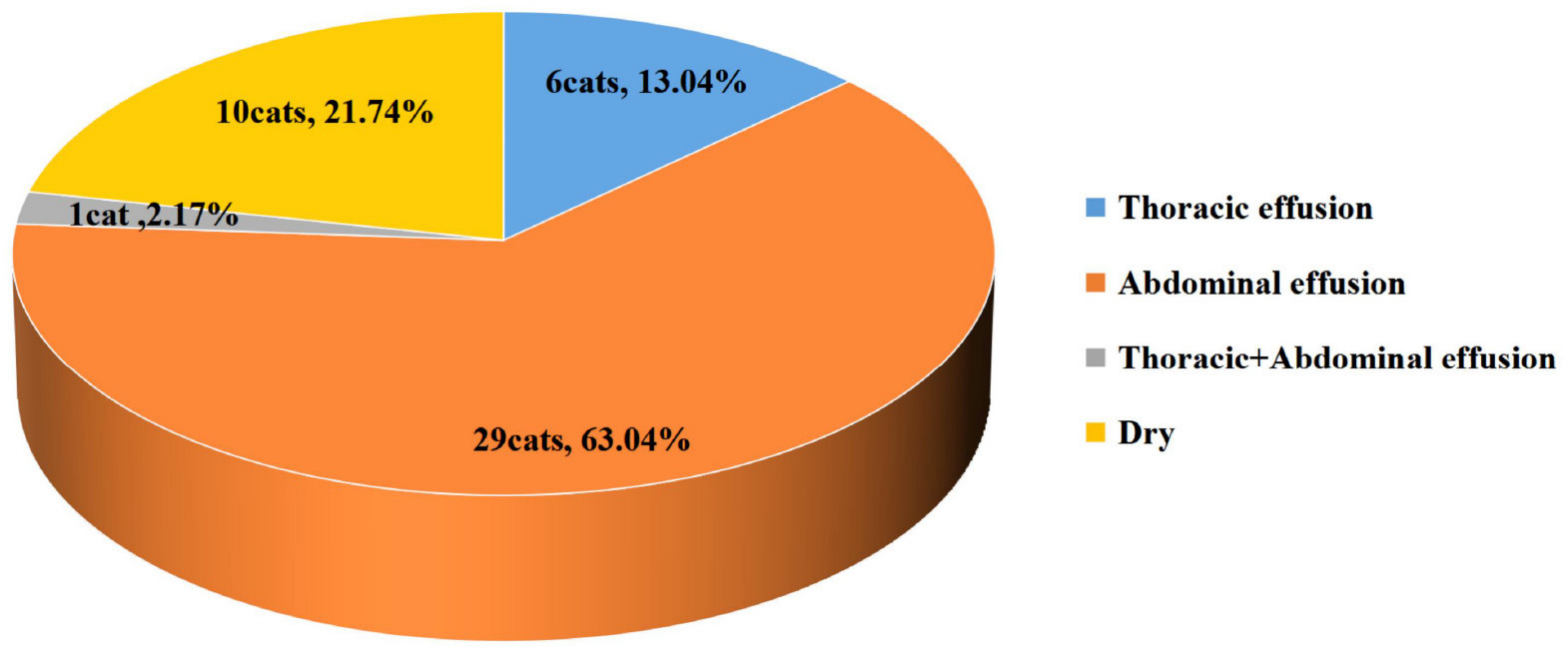
The pie chart shows the number and proportion of different clinical forms of infectious peritonitis (FIP) in cats from the 4 groups.

The types of thoracic+abdominal effusion in the affected cats are shown in Figure 2. The highest percentage of cats had abdominal effusion (63.04%), followed by dry-type disease (21.74%), thoracic effusion (13.04%), and thoracic+abdominal effusion (2.17%).

### General description

After combined drug treatment for 4 weeks, the observational data indicated that due to the severe course of the disease and the presence of serious complications at the beginning of treatment, cat CF2-3 died within the first treatment cycle (day 2 of treatment). Cats CF1-2 and CF1-7 were not clinically normal at the end of a 4-week treatment, and some indicators had not yet reached the criterion for discontinuation, so the owners requested continued treatment with GS-441524 alone. The drug was finally withdrawn after further treatment for 4 weeks and 7 weeks, respectively. The remaining cats in the group showed a good response to the combined drug treatment and were clinically normal by the end of the 4-week treatment period. At the time of writing, all 45 cats were alive and well, and no relapse had been observed.

The overall response of the enrolled cats to treatment was good, and their clinical signs improved within 24–48h from commencement of treatment. Their body temperature decreased, appetite and mental status improved, dehydration improved, and skin elasticity was restored. The post-treatment review indicated that 70% of the cats had gradually recovered their fur color after treatment for 2 weeks, and thoracic+abdominal effusion disappeared in 80% of the cats within 1–2 weeks of starting treatment. The mesenteric lymph nodes gradually decreased in size and returned to normal size, and perirenal edema eased and disappeared slowly. None of the cats showed any obvious skin reaction to the injection during treatment, although a small number of cats showed slight pain during the injection. After treatment, the white blood cell (WBC), lymphocyte (LYM), and TP levels decreased to the reference intervals, ALB increased, GLOB decreased, and A:G increased slowly. The jaundice symptoms of the cats gradually subsided within 2 weeks, and the liver enzyme indices, including aspartate acyltransferase (AST), alanine aminotransferase (ALT), TBIL, and γ-glutamyltransferase (GGT), gradually returned to the reference intervals after treatment for 4 weeks.

At the end of treatment, the cats in all four groups were clinically normal, the overall survival rate was 97.8% (CF2-3 death), although there were some differences in the specific recovery rates and changes in clinical indices (described below).

### Body temperature

**Figure 3:**
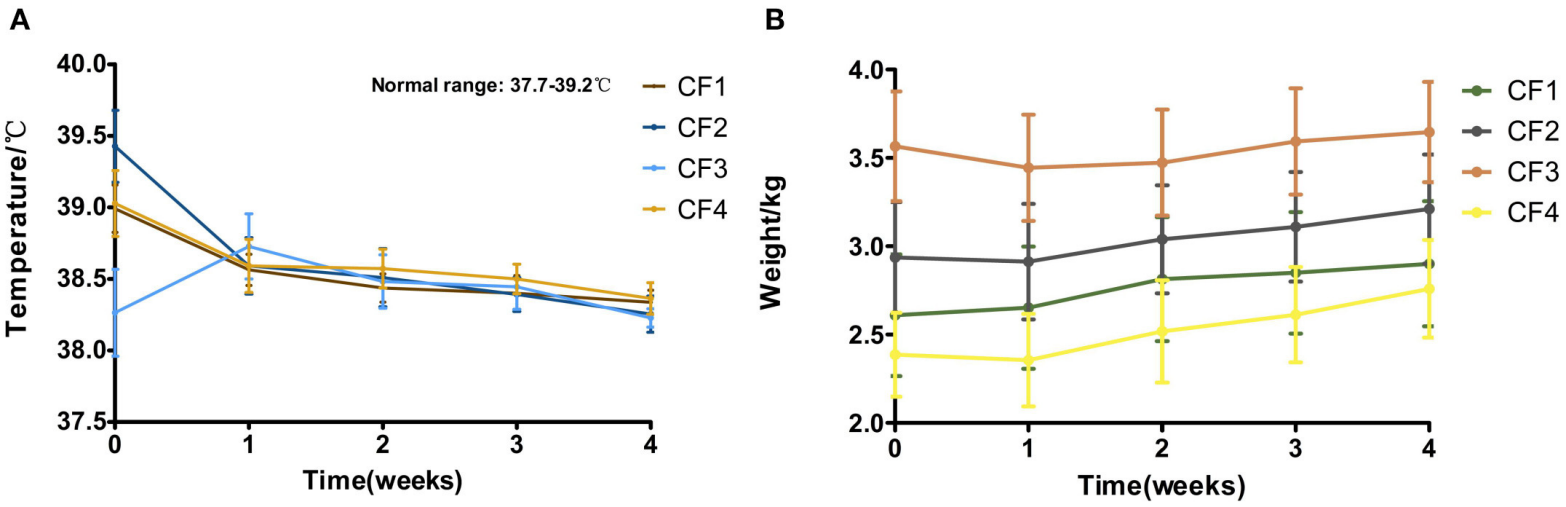
Mean body temperature, body weight variation and standard error for 4 groups cats that completed 4 weeks of treatment. **(A)** Body temperature. **(B)** Body weight.

The body temperature of most affected cats improved 2–3 days after treatment. However, the body temperatures of cats CF1-7, CF2-2, CF3-1, CF3-7, CF3-10, and CF4-11 increased within the first week of treatment, that of CF3-1 increased from 36.5 °C initially to 39.5 °C, and that of CF3-10 increased to 39.9 °C after treatment. The temperatures of the remaining cats increased by about 1 °C, but the average temperature of the cats gradually decreased to normal (37.7 to 39.2 °C) after treatment for 1 week. Except for the six cats with elevated body temperatures described above, the body temperatures of the other cats gradually returned to normal—here in other places. Within 72 h of the start of treatment. The changes in body temperature of all 46 cats are shown in Figure 3A.

### Body weight

The experiment was conducted in a randomized manner, so the ages of the cats enrolled in the experiment varied greatly, ranging from a minimum of 3 months to a maximum of 7 years, and their weights also varied greatly, from a minimum of 0.95 kg to a maximum of 5.7 kg. The increase in individual body weight is the most direct way to determine the growth of cats, and a positive change reflected the benign response of the cats to the drugs. After 1 week of treatment, most of the affected cats lost weight with the decrease of abdominal effusion or thoracic effusion. But in later treatment, the average weight of the CF1 group of cats maintained an increasing trend during treatment, with average weekly growth of > 10%. The average body weights of the CF2, CF3, and CF4 groups decreased slightly at the beginning of treatment, but increased slowly after the first week of treatment. The average body weight of the CF3 group increased relatively slowly (Figure 3B).

### WBC count

**Figure 4:**
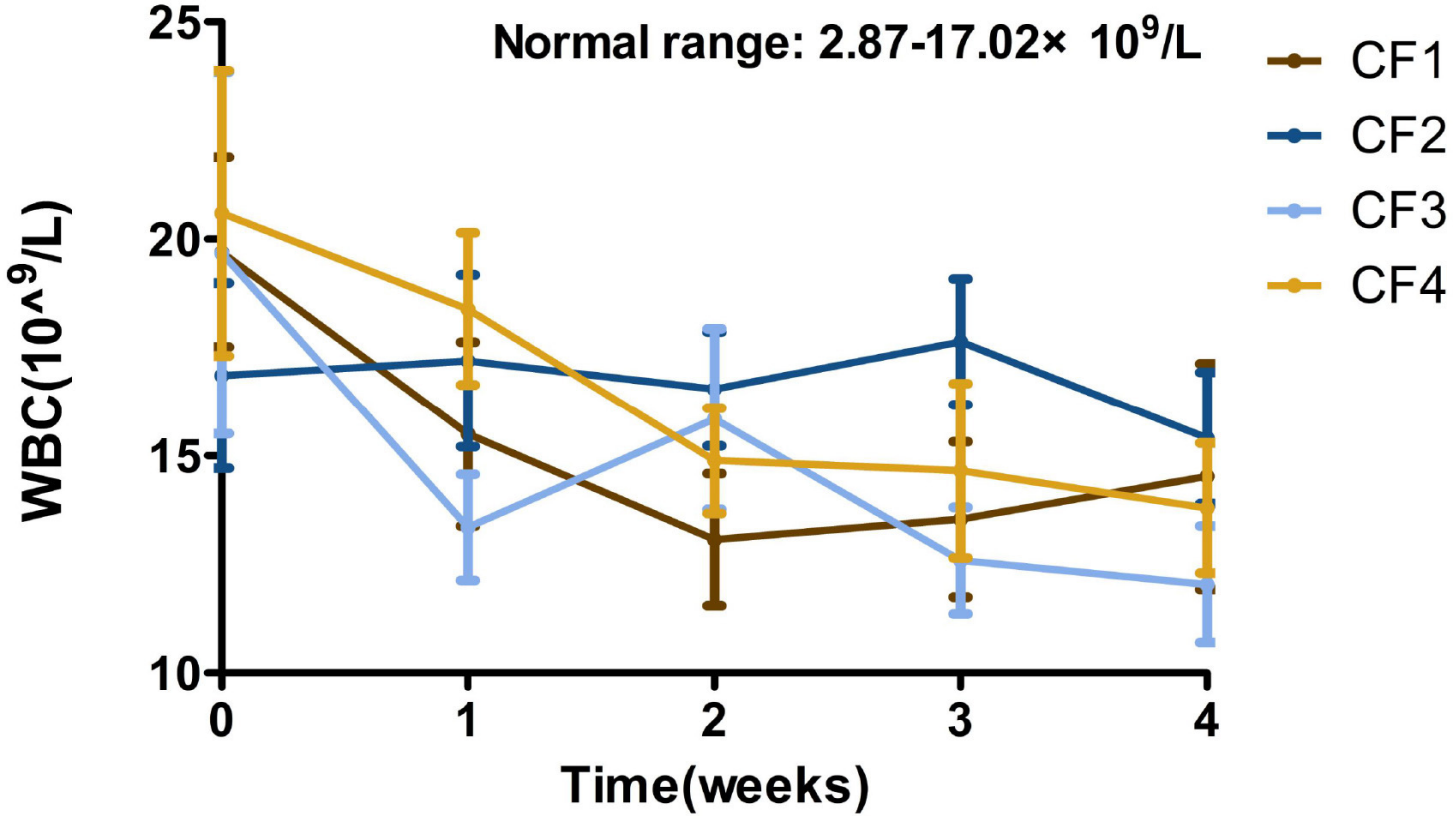
Mean WBC changes and standard error for 4 groups cats that completed 4 weeks of treatment. WBC, white blood cell.

Statistical data showed that the overall WBC levels were elevated in all the enrolled cats at the beginning of treatment and began to fall within 3–5 days of the treatment. The overall levels returning to within the reference interval after treatment for 1 week. The values in the CF3 group fluctuated significantly during the experiment, but recovered in the later stages of ongoing treatment (Figure 4).

### HGB and HCT

**Figure 5:**
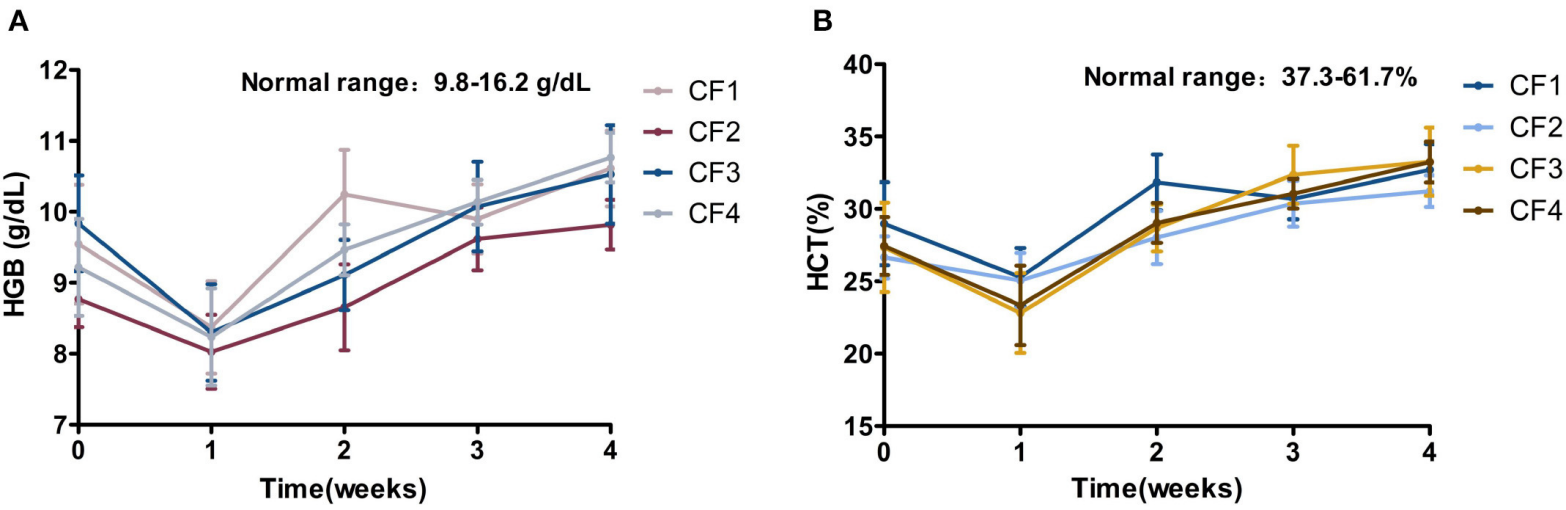
Mean HGB, HCT variation and standard error for 4 groups cats that completed 4 weeks of treatment. **(A)** HGB. **(B)** HCT. HGB, hemoglobin; HCT, hematocrit.

The HGB index was at the lower limit of the reference interval or slightly below it at the beginning of treatment, and HCT was below the reference interval in all four groups. After treatment for 1 week, the HGB and HCT indices of all the cats had decreased significantly relative to baseline, suggesting that the anemia of the cats worsened within the first week of treatment. After treatment for 2 weeks, the values of the two indices started to increase, and those of group CF1 increased most significantly. At the end of the 4-week treatment period, HGB had returned to the reference interval, whereas HCT had not, although there was a unstable upward trend in its value in all groups (Figure 5).

### Serum protein changes (TP, ALB, and GLOB)

**Figure 6:**
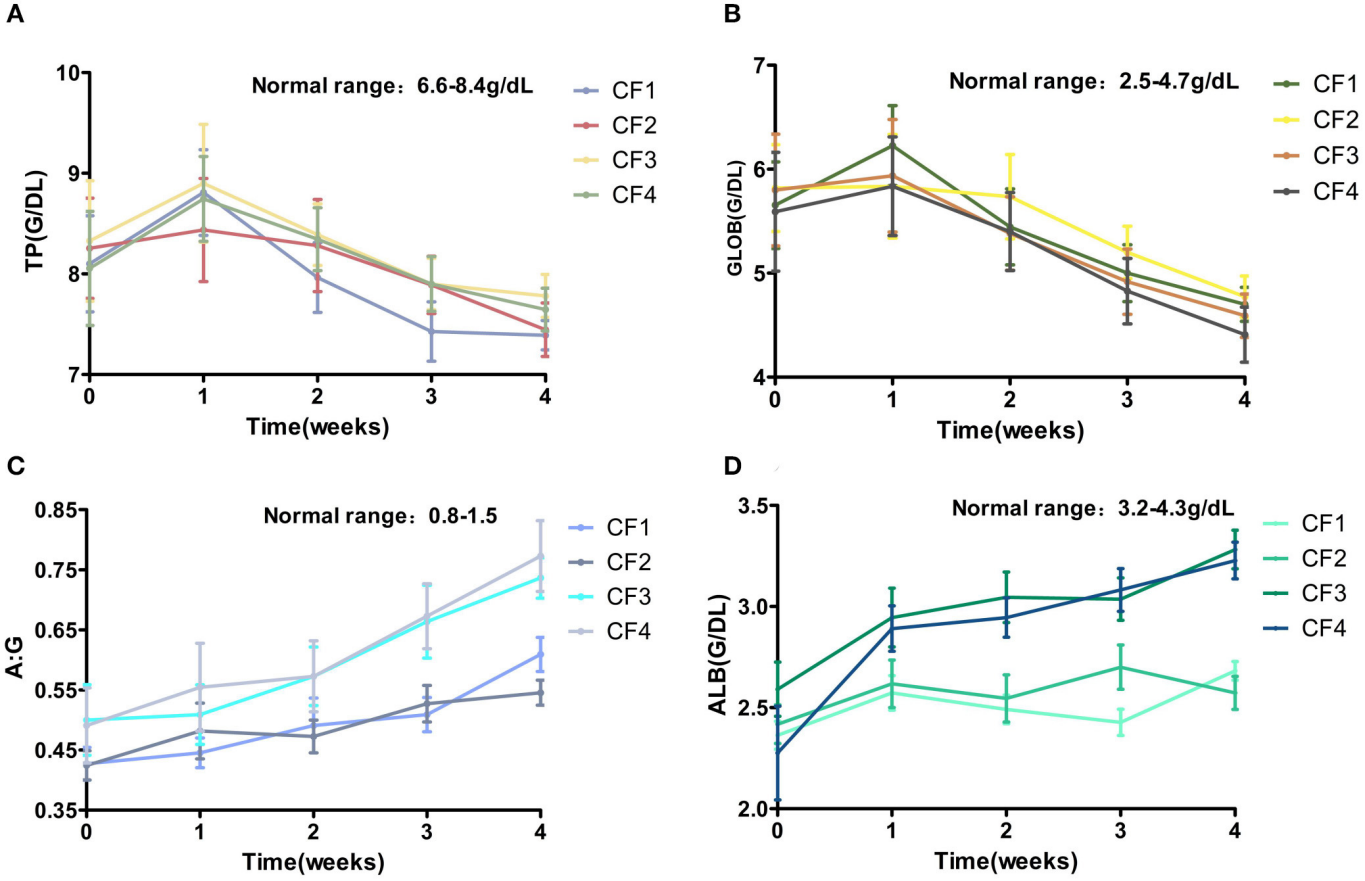
Mean TP, GLOB, ALB, A:B changes and standard error for 4 groups cats that completed 4 weeks of treatment. **(A)** TP. **(B)** GLOB. **(C)** ALB. **(D)** A:G. TP, total protein; GLOB, globulin; ALB, albumin; A:G, albumin–globulin ratio.

The ALB levels of all the cats decreased significantly, the GLOB level increased, and the A:G ratio decreased significantly. The A:G ratio of cat CF3-10 was only 0.2 before treatment. The TP and GLOB levels of this cat continued to increase during 1 week treatment and reached a peak, and the amount of fluid in the cat's abdomen decreased. After treatment for 2 weeks, CF3-10 TP and GLOB levels began to decrease significantly and fell to the highest critical value (3.7 g/dL) by the third week of treatment. Its ALB levels and A:G continued to increase. The change in A:G played a crucial role in the treatment of all the affected cats, and the average A:G ratio continued to increase slowly throughout the treatment period (Figure 6).

### TBIL

**Figure 7:**
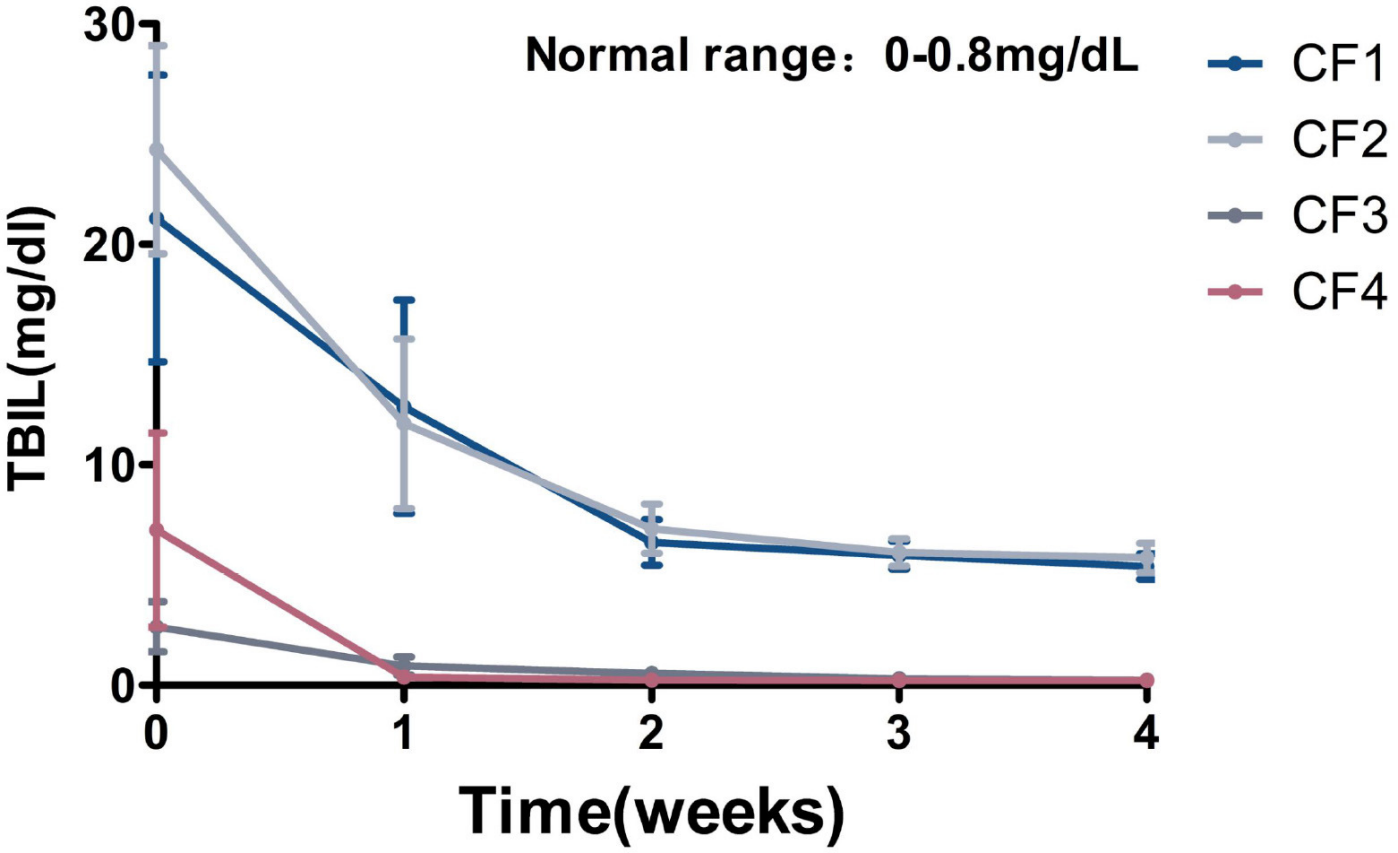
Mean TBIL changes and standard error for 4 groups cats that completed 4 weeks of treatment. TBIL, total bilirubin.

In the early stage of FIP, most of the cats had overt jaundice, and biochemical tests revealed abnormal liver function indices, some of which were more than 10-fold higher than normal, including TBIL, ALT, AST, and GGT. All TBIL values were significantly abnormal, and most exceeded the reference interval 10–40-fold (reference interval, 0–0.8 mg/dL). This suggests that FCoV damages the liver function of affected cats, so it is important to monitor the liver function indices during treatment. When the cats were reviewed after treatment for 1 week, all their liver function indices had decreased significantly, and had returned to the reference intervals in some cats. Taking TBIL as an example, as shown in Figure 7, the TBIL levels of all cats decreased significantly when reviewed after treatment for 1 week. The initial TBIL levels of the CF1 and CF2 cats exceeded the normal upper limit 30-fold, but had decreased significantly after treatment for 1 week. In groups CF3 and CF4, the index returned to within the reference interval after 1 week of treatment, and no further abnormalities were observed (Figure 7).

## Discussion

A review of the published literature showed that GS-441524 and GC376 were successfully used for the treatment of FIP when used alone (7, 8, 14). The two drugs have different mechanisms of antiviral action (1, 16). GS-441524 mainly acts in the early stages of viral replication, and competitively inhibits the replication of coronavirus RNA, thus, terminating viral replication (12). In contrast, GC376 targets the 3C protease of coronavirus and prevents the protease from synthesizing the viral capsid proteins, thus, inhibiting viral assembly (17). In an early study, GS-441524 was more effective than GC376 in the treatment of cats with FIP (10). The remission rate with GC376 was about 50%, whereas that with GS-441524 was 70% (18). Based on that study, we hypothesized that the combination of GC376 and GS-441524 might produce an additive therapeutic effect. The results of the present study supported our hypothesis, and the overall treatment remission rate with GS-441524 combined with GC376 was 93.5%, which far exceeded the results of either drug used alone. The overall recovery and post-treatment relapse rates in the affected cats were also significantly better after the combination therapy than after either drug alone. Our data provide a reliable reference for the clinical use of the two drugs and the development of appropriate treatment protocols.

An important finding of this study was the dramatic reduction in the treatment period in comparison with those reported in the literature for each drug alone, GC376 alone required administration for 12–17 weeks (7) and GS-441524 alone required 12 weeks (8, 19), whereas the combination of GC376 and GS-441524 dramatically reduced the treatment period to 4 weeks. This was undoubtedly of benefit for the affected cats and their owners. However, some clinical indicators had not returned to the reference intervals by the end of the experiment. For instance, the A:G ratio was slightly below normal for cats in the CF1 and CF2 groups. The failure of these indicators to reach the reference intervals did not affect the return of the cats to health, and no relapse occurred.

There were four treatment groups in this experiment (Table 2). The doses of GC376 and GS-441524 were determined with reference to the pharmacokinetics of the two drugs in the experimental cats and the relevant clinical experimental data (12, 18). After treatment, the data and clinical performances showed that the four groups of cats responded well to treatment. This may be related to the fact that the combination of the drugs inhibited the virus at different stages of replication and controlled it effectively within a short period of time. Varying degrees of resistance to both drugs have been reported when used alone (3, 5). In studies of GS-5734, the esistance to GS-441524, the precursor drug of GS-5734, was also detected (20). In that experiment, cats were given two injections of GC376 and one injection of GS-441524 subcutaneously each day, and most cats showed only mild injection pain at the time of injection. No injection reaction, such as local erythema, subcutaneous tissue nodules, or necrosis, was observed during treatment. Previously, some investigators have reported mild adverse reactions after adjusting the pH was adjusted to 1.5 after the drug was diluted, indicating that a too-low pH may lead to painful injection site reactions or even inflammatory reactions at the injection site in animals (7, 8). We adjusted the pH value of the drug to 3–4, thus avoiding these problems. The mild local injection reactions in the present study confirmed that the combination of the two drugs is safer than either drug alone, and provides a reference for later clinical trials of the drugs.

Close monitoring each indicators of cats with FIP during their treatment is important in understanding the course of the disease and its timely control. In this study, we closely monitored several indicators, including body temperature, body weight, WBC, HCT, HGB, TP, GLOB, ALB, A:G, and TBIL. During the 4-week experiment, the routine hematology and serum biochemistry indices of most of the FIP-affected cats improved significantly and approached the reference intervals. However, some indices had not returned to normal at the end of treatment. When the disease is accompanied by renal lesions or lymph-node enlargement, a longer recovery time may be required. The clinical complexity of FIP is also illustrated by the production of large amounts of chemokines, cytokines, adhesion factors, selectins, and integrins in response to inflammatory granuloma-like changes in the abdominal cavity, which may require longer treatment cycles or higher drug doses (21).

Our statistical data showed differences in some of the test results for the four dosage groups, which may be related to the dose of the drugs and individual differences. For example, the rate of disappearance of abdominal effusion was higher in the CF1 and CF2 than in the CF3 and CF4 group, and the changes in body weight gain were smaller in the CF3 than in the other three groups. However, the other blood biochemical parameters did not change with any regularity, and when all the experimental data are considered, the most important reason for the differences in blood biochemical parameters may be individual differences. The experiment also revealed some interesting data regarding, for example, HCT and HGB. The values for HCT were at the lower limit of the reference interval when the CF1 and CF3 was first examined, and the values for HGB were within the lower limit of the reference interval or slightly below the reference interval. Both indices indicated that the cats were in a mildly anemic state. However, when the cats were examined after treatment for 1 week, the values for HCT and HGB in all four groups had decreased significantly, but increased after 2 weeks of treatment. We inferred that the cat's anemia may have been relatively serious when they were enrolled in the group, and that most of the cats were severely dehydrated in the early stage of treatment, causing the artifact of a relatively high HCT index. The slight anemia artifact caused by other factors disappeared, and the degree of anemia in the cats may have improved or remained in the original state. However, this cannot be accurately assessed due, again to the effect of dehydration in the early stage. This is only a hypothesis, but we hope to test it in future research.

In this study, controlling FCoV infection at different points in the viral replication process using GS-441524 combined with GC376 reduced the treatment period and improved efficacy of treatment in comparison with data reported for each individual drug in previous studies (7, 8). These results may also be relevant for the consideration of the treatment options for people infected with SARS-CoV, consistent with the conjecture of Paltrinieri et al. (2).

## Conclusions

In this study, we showed that the combination of GS-441524 and GC376 was effective in the treatment of FIP-affected cats, confirming the hypothesized additive effect of the drugs. The 4-week treatment period was considerably shorter than those describe previously for either drug when used alone. The dosing regimens and treatment parameters used in this experiment should facilitate the commercialization of both drugs in the future.

## Data availability statement

The original contributions presented in the study are included in the article/Supplementary material, further inquiries can be directed to the corresponding authors.

## Ethics statement

The animal study was reviewed and approved by China Agricultural University regulations concerning protection of animals used for scientific purposes. Written informed consent was obtained from the owners for the participation of their animals in this study.

## Author contributions

JD, JL, and YJ conceived the study. JD, JL, YB, YW, and LY performed the experiments. JD, JL, YB, and YW analyzed the data and wrote the manuscript. All authors have read and approved the final version of the manuscript.

## Conflict of interest

The authors declare that the research was conducted in the absence of any commercial or financial relationships that could be construed as a potential conflict of interest.

## Publisher's note

All claims expressed in this article are solely those of the authors and do not necessarily represent those of their affiliated organizations, or those of the publisher, the editors and the reviewers. Any product that may be evaluated in this article, or claim that may be made by its manufacturer, is not guaranteed or endorsed by the publisher.
