# Supplementary material for: Effect of GS-441524 in combination with the 3C-like protease inhibitor GC376 on the treatment of naturally transmitted feline infectious peritonitis
Source: Front Vet Sci. 2022 Oct 28;9:1002488. doi: 10.3389/fvets.2022.1002488 (PMC9650422; doi:10.3389/fvets.2022.1002488)
Supplement: Supplementary file 1 [file Data_Sheet_1.PDF]

Supporting information for *Frontiers Veterinary Science*

Effect of GS-441524 in combination with 3C-like protease inhibitor GC376 on the treatment of naturally infected feline infectious peritonitis

Jinbao Lv1, Yang Bai1, Yingyun Wang1, Liu Yang1, Yipeng Jin1, *, Jun Dong 1, *

1 College of Veterinary Medicine, China Agricultural University, Beijing, China

*** Correspondence:**
Corresponding Author: Jun Dong and Yipeng Jin
email: vetdong@163.com (J. Dong), yipengjin@sina.com (Y.P. Jin).

Corresponding author:
J. Dong, senior veterinary physician, PhD, Y.P. Jin, Professor, College of Veterinary Medicine, West Campus of China Agricultural University, No. 2 Yuanmingyuan West Road, Haidian District, CA100193, Beijing, Email: vetdong@163.com (J. Dong).

Contents:

Table 1. Body Weight

Table 2. Body Temperature

Table 3. HCT (hematocrit)

Table 4. HGB (hemoglobin)

Table 5. WBC (white blood cell)

Table 6. TBIL (total bilirubin)

Table 7. GLOB (globulin)

Table 8. ALB (albumin)

Table 9. TP (total protein)

Table 10. A:G (albumin–globulin ratio)

Table 1 Body Weight

Group	0W	1W	2W	3W	4W
CF1-1	3.38	3.60	4.10	4.17	4.30
CF1-2	1.30	1.43	1.73	2.00	2.10
CF1-3	3.40	3.60	3.60	3.60	3.65
CF1-4	4.16	4.38	4.55	4.44	4.61
CF1-5	2.60	2.42	2.50	2.44	2.35
CF1-6	0.95	0.94	1.10	1.10	1.10
CF1-7	1.95	1.90	1.90	2.00	2.05
CF1-8	4.30	4.10	4.20	4.30	4.30
CF1-9	1.40	1.63	1.72	1.70	1.75
CF1-10	2.96	2.90	3.00	3.00	3.10
CF1-11	2.30	2.28	2.55	2.60	2.60
CF2-1	3.65	2.80	3.00	3.40	3.75
CF2-2	3.00	2.90	3.20	3.10	3.10
CF2-3	3.61	4.10	4.10	4.20	4.20
CF2-4	2.80	2.80	3.00	2.80	3.30
CF2-5	4.00	4.10	4.00	4.10	4.10
CF2-6	1.60	1.60	1.79	1.70	1.80
CF2-7	1.90	1.80	1.85	1.98	2.00
CF2-8	3.40	3.50	3.60	3.70	3.70
CF2-9	1.64	1.54	1.84	1.93	1.92
CF2-10	4.70	4.70	4.75	4.80	4.85
CF2-11	2.00	2.20	2.30	2.50	2.60
CF3-1	2.70	2.50	2.30	2.40	2.50
CF3-2	3.30	3.60	3.90	4.30	4.30
CF3-3	3.56	3.43	3.48	3.59	3.65
CF3-4	3.15	3.05	3.07	3.33	3.36
CF3-5	3.50	3.40	3.50	3.50	3.70
CF3-6	3.50	3.30	3.30	3.50	3.60
CF3-7	4.06	3.94	3.79	4.25	4.30
CF3-8	2.91	2.75	3.10	3.15	3.20
CF3-9	3.80	3.60	3.80	3.80	3.90
CF3-10	1.85	1.70	1.60	1.60	1.70
CF3-11	4.75	4.85	4.70	4.60	4.75
CF3-12	5.70	5.20	5.15	5.10	4.80
CF4-1	2.45	2.43	2.43	2.49	3.10
CF4-2	1.10	1.00	1.00	1.30	1.50
CF4-3	3.00	2.80	3.10	2.90	3.10
CF4-4	2.40	2.55	2.50	2.60	2.70
CF4-5	2.56	2.65	2.85	3.00	3.10
CF4-6	2.85	3.15	3.50	3.65	3.70
CF4-7	1.22	0.90	1.48	1.75	1.70
CF4-8	3.60	3.53	3.84	3.90	4.05
CF4-9	2.00	2.00	2.00	2.10	2.20
CF4-10	1.87	1.70	1.40	1.45	1.50
CF4-11	3.20	3.20	3.60	3.60	3.70
CF4-12	2.10	2.31	2.35	2.66	2.70

Table 2 Body Temperature

Group	0W	1W	2W	3W	4W
CF1-1	39.6	38.3	38	38.4	38.5
CF1-2	39.2	38.9	38.6	37.8	38
CF1-3	38.6	38.6	38.2	38.3	38.3
CF1-4	39.4	38.4	38.2	38.3	37.8
CF1-5	39.1	39.1	38.5	38.5	38.4
CF1-6	39	38.3	38.2	38.2	38.2
CF1-7	37.8	39.1	38.9	39	38.8
CF1-8	39.6	38.7	38.2	37.8	38.2
CF1-9	39.4	38.1	38.3	39.1	38.5
CF1-10	38.6	38.1	38.7	38.6	38.6
CF1-11	38.6	38.6	39	38.4	38.4
CF2-1	40.1	39.8	39.7	38.8	38.6
CF2-2	38.1	39.4	38.3	38.6	38
CF2-3	39.5	38.1	38.2	38.4	37.8
CF2-4	38.7	38.5	38.3	38.3	38.5
CF2-5	40.2	38.2	38	38.1	38
CF2-6	39.5	39.3	39.4	38.8	38.6
CF2-7	39.1	38.7	39.1	39.1	38.7
CF2-8	38.7	38.2	38.4	38.2	38.5
CF2-9	39.9	38.4	38.6	38.2	38.6
CF2-10	38.9	38.3	38.3	38	38.1
CF2-11	41	37.6	37.3	37.8	37.4
CF3-1	36.5	39.5	38.6	38.2	38.1
CF3-2	38.3	38.7	38.5	38.4	38.2
CF3-3	39	39	38.6	38.5	38.5
CF3-4	39	38.4	39.6	39.5	38
CF3-5	38.1	38.5	38.5	38.6	38.4
CF3-6	38	38.3	38.2	38.1	38.3
CF3-7	38.6	39.6	38.5	38.4	38.2
CF3-8	38.5	38.3	38.4	38.3	38.3
CF3-9	40.3	38.5	38.3	38.5	38.1
CF3-10	38.1	39.9	39.2	39.1	38.6
CF3-11	37.4	37.2	37.1	37.5	38.1
CF3-12	37.4	38.8	38.3	38.2	37.9
CF4-1	38.6	38.2	38.3	38.2	38.5
CF4-2	39.2	38.6	39	39	38.5
CF4-3	38.1	38	38.4	38.2	38.3
CF4-4	39.5	39.6	39.2	38.7	38.6
CF4-5	39.4	39.2	38.3	38.4	38.3
CF4-6	39.8	38.1	38.2	38.1	38.2
CF4-7	39.5	38	38	38.5	37.4
CF4-8	38.5	37.9	38.1	38	38.3
CF4-9	39.2	39	39.2	38.8	38.8
CF4-10	37.5	39.4	38.6	38.7	38.5
CF4-11	40	38.5	39	38.9	38.6
CF4-12	38.9	38.5	38.4	38.6	38.4

Table 3 HCT

Group	0W	1W	2W	3W	4W
CF1-1	29.6	23.2	28.5	29.4	34.3
CF1-2	21.4	23.1	22.2	22.9	23.7
CF1-3	33.3	27.7	38	33.2	39.3
CF1-4	49.3	38.7	41	34.2	39.3
CF1-5	28.6	29.9	32.1	36.4	37.5
CF1-6	25.4	21.3	22.1	24.8	25.2
CF1-7	16.3	15.8	25.9	25.6	25.8
CF1-8	37.5	26.1	36.9	28.6	28.2
CF1-9	16.6	21.4	33.4	32.4	33.7
CF1-10	27.7	18.4	33.6	35	37.6
CF1-11	33.1	32.6	36.4	35	35.1
CF2-1	22.8	14.6	19.1	23.9	26.2
CF2-2	27.5	25.3	24.2	29.8	34
CF2-3	21.9	17.4	21.2	20.8	25.4
CF2-4	27.1	25.3	24.8	29.2	28.4
CF2-5	28.6	25.6	36.9	33.4	35.3
CF2-6	31.8	18.8	26.6	28.7	32.5
CF2-7	28.7	33.1	31.4	38.3	33
CF2-8	29.2	35.2	32.8	32.7	35.6
CF2-9	23.6	25.7	24.9	27.1	27.9
CF2-10	34.4	29.6	37.5	36.6	32.7
CF2-11	17.5	24.9	28.8	33.4	32.4
CF3-1	38.3	28.3	28.6	28.2	42
CF3-2	31.2	32.2	37.8	42.8	42.2
CF3-3	24.24	27.7	33	30	30.2
CF3-4	33.63	3.43	21.4	27.2	30.8
CF3-5	30.9	29	30.3	30.3	26.6
CF3-6	21.3	22.6	25.3	25.7	22.6
CF3-7	3.46	21.5	29.2	33	45.9
CF3-8	18.7	15.1	28.2	36	37.7
CF3-9	33.8	30.8	30.7	30.9	32.5
CF3-10	26.5	11.7	18.6	26	23.4
CF3-11	38.7	28.6	32.4	45.8	31.9
CF3-12	35.7	34.6	23.2	28.7	36.2
CF4-1	33	27.3	21.2	27.7	28.4
CF4-2	20.5	13.4	27.5	33.3	28.6
CF4-3	33.6	32.6	38.3	33.7	42
CF4-4	30.5	33.6	26.4	31	35.7
CF4-5	26.8	23.4	26.1	28.5	31.5
CF4-6	16.4	21.8	27.6	29.1	31.7
CF4-7	23.5	1.48	32.1	32	38.3
CF4-8	22.6	23.9	31.8	31.4	32.6
CF4-9	39.6	28.3	32.7	33.3	34
CF4-10	27	23.7	25.8	24.6	26.4
CF4-11	28.2	27.3	29.8	36.9	36.3
CF4-12	31.9	32.1	34.5	35.3	33.5

Table 4 HGB

Group	0W	1W	2W	3W	4W
CF1-1	10.2	8	9.5	9.3	10.5
CF1-2	7.3	7.7	7.1	7.5	7.7
CF1-3	10.5	9.1	11.9	10.4	11.7
CF1-4	15.4	12.7	13	10.7	12.2
CF1-5	9.6	9.7	10.3	11.7	11.7
CF1-6	7.8	6.7	7.2	8	8.5
CF1-7	6.2	5.1	8	7.9	8.5
CF1-8	12.1	8.7	11.9	9.3	9.5
CF1-9	5.8	7.2	10.6	10.6	11.4
CF1-10	9.4	6.4	10.6	11.2	12.6
CF1-11	10.7	10.8	12.6	12.3	12.4
CF2-1	7.7	5.1	6	7.4	8
CF2-2	9.7	8.7	8.2	9.7	10.8
CF2-3	8	6.1	7.1	6.9	8.1
CF2-4	8.5	7.7	8.2	9.1	8.9
CF2-5	8.9	8.1	11.8	10.9	11.5
CF2-6	9.9	6.4	5.6	9.6	9.9
CF2-7	8.9	9.9	9.6	11.7	10.1
CF2-8	10.2	11.2	10.8	10.3	11.2
CF2-9	7.9	8.2	8.1	9	9.2
CF2-10	10.7	9	11	10.9	10.1
CF2-11	6.1	7.9	8.8	10.3	20.2
CF3-1	12.2	9.3	8.1	8.1	12
CF3-2	9.9	10.3	12.2	13.3	13.3
CF3-3	7.9	8.2	9.5	8.7	8.7
CF3-4	11.8	11	7.8	8.9	10.1
CF3-5	9.9	9.4	9.6	9.7	8.8
CF3-6	7.6	7.3	8.1	8	7.5
CF3-7	11.1	7.3	9.4	10.3	14.8
CF3-8	5.7	5.3	8.3	10.3	11
CF3-9	10.9	10.4	10.3	10.9	11.4
CF3-10	8.3	3.7	6.2	8.3	7.7
CF3-11	12.9	9.1	10.7	14.3	10.5
CF3-12	10.5	10.7	7.5	8.2	10.5
CF4-1	11.9	9.5	7.7	9	9.6
CF4-2	6.5	4.3	8.2	9.5	8.7
CF4-3	10.3	9.8	11.3	10.2	12.2
CF4-4	9.8	11	8.8	9.8	11
CF4-5	9.1	8.1	8.5	8.9	9.9
CF4-6	5.5	7.2	8.8	9.1	10
CF4-7	8.1	4.4	9.6	10.5	12
CF4-8	7.3	7.4	9.9	9.8	10.3
CF4-9	13	9.5	10.7	11.4	11.7
CF4-10	9.7	8.2	9	7.8	10.7
CF4-11	9.1	8.8	9.5	11.5	12
CF4-12	10.8	10.6	11.1	11.8	11

Table 5 WBC

Group	0W	1W	2W	3W	4W
CF1-1	19.21	16.73	15	14.72	18.19
CF1-2	16.7	14.54	12.04	11.41	9.62
CF1-3	24.78	9.65	8.82	10.15	8.47
CF1-4	30.49	24.69	19.98	25.32	36.25
CF1-5	20.05	13.72	18.51	20.66	21.31
CF1-6	9.05	7.75	9.47	8.05	7.68
CF1-7	27.5	19.27	17.67	20.23	16.5
CF1-8	10.06	19.44	18.92	10.69	15.54
CF1-9	13.97	9.61	6.67	10.88	9.56
CF1-10	27.85	28.52	8.64	9.59	8.34
CF1-11	17	6.61	8	7.2	8.3
CF2-1	26.34	24.79	20.24	19.27	17.08
CF2-2	8.72	13.45	16.08	11.12	14.46
CF2-3	9.28	4.8	18.61	27.7	28.85
CF2-4	16.51	17.48	26	17.2	17.53
CF2-5	12.3	12.61	13.9	16.95	13.6
CF2-6	28.28	23.29	14.29	25.05	10.57
CF2-7	10.2	27.1	12.31	15.75	11.36
CF2-8	16.83	14.02	17.64	15.66	13.56
CF2-9	21.8	16.75	19.27	15.15	16.92
CF2-10	11.77	12.87	11.84	13.6	11.7
CF2-11	23.35	21.95	11.81	16.5	13.89
CF3-1	14	9.63	27.39	16.62	10.93
CF3-2	17.3	12.81	9.75	9.46	8.34
CF3-3	18.22	18.62	18.35	20.11	22.19
CF3-4	14.3	8.82	8.57	9.41	10.27
CF3-5	5.89	12.52	12.54	7.46	12.51
CF3-6	13.51	8.6	7.77	9.56	11.32
CF3-7	33.68	13.27	26.7	13.91	13
CF3-8	55.57	20.69	13.17	13.23	12.48
CF3-9	9.69	16.87	20.21	17.6	16.3
CF3-10	19.75	14.82	18.58	9.8	4.81
CF3-11	14.5	10.23	11.3	11.29	10.28
CF3-12	26.57	13.5	14.18	10.62	6.76
CF4-1	11.34	19.61	12.37	4.47	6.16
CF4-2	14.21	21.66	10.25	7.73	10.69
CF4-3	19.75	15.98	14.36	18.19	15.81
CF4-4	22.47	15.04	8.64	18.96	8.22
CF4-5	28.76	22.88	18.01	21.04	19.47
CF4-6	7.33	13.09	14.1	10.74	11.55
CF4-7	18.2	27	21.24	12	12.83
CF4-8	10.9	12.79	20.81	26.47	23.39
CF4-9	41.4	27.88	12.15	11.7	12.6
CF4-10	36.6	10.86	15.7	19.94	17.4
CF4-11	15.58	15.49	16.13	9.98	13.64
CF4-12	14.3	17.14	11.65	12.57	17.68

Table 6 TBIL

Group	0W	1W	2W	3W	4W
CF-1	33	6.6	6.1	3.5	6
CF-2	5	5	5.1	6	8
CF-3	10	9	4.1	7	4.4
CF-4	2.8	4.1	5	4.9	2.9
CF-5	3.1	2.9	2.7	4	4.8
CF-6	19.4	8.1	4	4.4	3.7
CF-7	16	12	7	10	8
CF-8	11.4	11	8	8	4.3
CF-9	10.2	9	6.6	8	7
CF-10	57	11	6.9	4	3
CF-11	65.1	60.2	15.7	5.1	7.1
CF2-1	32.5	49.5	8.4	2.5	6
CF2-2	19	5.8	9	9	5
CF2-3	45	10	15.5	6.2	9
CF2-4	39.5	9	3.8	6.4	2
CF2-5	20	10	1.6	7	7.6
CF2-6	9	8.5	10	5.3	5
CF2-7	10.79	8	6	3.2	7.7
CF2-8	46	5	6.7	9	7
CF2-9	4.9	4	6.1	5.7	6.2
CF2-10	6.5	7.9	7	7	6
CF2-11	34	12.8	4	4.9	2
CF3-1	4.6	3.4	2.1	1	0.5
CF3-2	0.3	0.2	0.2	0.2	0.2
CF3-3	6	0.2	0.2	0.2	0.2
CF3-4	12	0.6	0.2	0.2	0.2
CF3-5	0.5	0.2	0.2	0.2	0.2
CF3-6	0.2	0.2	0.2	0.2	0.2
CF3-7	4	3.8	1.7	0.5	0.2
CF3-8	0.6	0.2	0.2	0.2	0.2
CF3-9	0.2	0.2	0.3	0.2	0.2
CF3-10	0.2	0.2	0.2	0.2	0.2
CF3-11	0.5	0.4	0.3	0.2	0.2
CF3-12	3.2	0.7	0.3	0.2	0.2
CF4-1	0.5	0.5	0.2	0.2	0.2
CF4-2	0.5	0.2	0.2	0.2	0.2
CF4-3	0.2	0.2	0.2	0.2	0.3
CF4-4	0.2	0.2	0.2	0.2	0.2
CF4-5	0.2	0.2	0.2	0.2	0.2
CF4-6	4.4	1.4	0.5	0.3	0.2
CF4-7	0.6	0.2	0.2	0.2	0.2
CF4-8	0.2	0.2	0.2	0.2	0.2
CF4-9	26	0.2	0.2	0.2	0.2
CF4-10	44.5	0.5	0.3	0.2	0.2
CF4-11	0.2	0.2	0.2	0.2	0.2
CF4-12	27	0.3	0.2	0.2	0.2

Table 7 GLOB

Group	0W	1W	2W	3W	4W
CF1-1	4.5	5.1	6.2	5.9	5
CF1-2	4.5	4.3	4.1	3.6	4
CF1-3	8.8	7.2	6	4.8	4.6
CF1-4	5.6	5.8	3.4	3.8	4.3
CF1-5	4.9	8.7	7.5	6.4	4.8
CF1-6	4.1	5.6	4.1	3.9	3.8
CF1-7	6.3	6.1	6.2	5.6	5.4
CF1-8	4.7	7.3	6.5	4.9	4.3
CF1-9	5.9	6.5	5.5	5.2	5
CF1-10	5.8	4.9	5.3	5.6	5.4
CF1-11	7.1	7	5.1	5.3	5.1
CF2-1	6.1	6.1	5	5.2	5.2
CF2-2	5.5	9.9	9.2	7.2	6.3
CF2-3	7.3	4.7	4.5	4.6	3.6
CF2-4	3.6	3.5	5.2	4.5	4.3
CF2-5	7.9	6.1	5.8	5	4.6
CF2-6	7.5	7.2	7	5.8	5.1
CF2-7	5.5	5.3	5.1	5.8	4.6
CF2-8	3.8	5.6	5	5.1	4.7
CF2-9	5.4	4.7	4.9	5.1	4.6
CF2-10	5.3	5.9	6.2	4.8	4.9
CF2-11	6.1	5.2	5.2	4.1	4.6
CF3-1	5.6	5.6	6.1	5.9	5.7
CF3-2	9.3	9.2	6.3	5.9	4.8
CF3-3	7.5	7.2	6	5.3	4.6
CF3-4	6.8	5	4.8	5	4.3
CF3-5	4.8	5.3	5	4.3	4
CF3-6	3.7	3.9	4.1	3.7	3.8
CF3-7	4.7	4.2	4.6	3.6	4
CF3-8	3.2	5.1	4.3	3.8	4.1
CF3-9	6.2	6.9	6.1	6	5.7
CF3-10	7	8.6	7.8	6.4	5.3
CF3-11	6.2	7.7	7	4.2	4.2
CF3-12	5	4.3	4.1	4.2	4.2
CF4-1	6.4	6	5.5	5.5	5
CF4-2	4.8	6.1	5.3	4.2	3.7
CF4-3	6.2	5.2	4.8	3.8	4.6
CF4-4	4.9	6.9	6.5	5.5	3.4
CF4-5	5.4	6.8	7	5.6	5
CF4-6	5.2	5.5	4.7	4.4	4.1
CF4-7	6.9	6.4	5.2	4.9	4.9
CF4-8	9.7	8.6	7.7	7.1	6.4
CF4-9	2.6	3.2	3.9	3.8	3.8
CF4-10	3.2	3.2	3.6	3.6	3.6
CF4-11	6.2	6.3	5.2	4.7	4
CF4-12	6.1	6.2	5.7	5.1	3.9

Table 8 ALB

Group	0W	1W	2W	3W	4W
CF1-1	2.4	3	2.4	2.1	2.8
CF1-2	1.9	2	2.2	2.2	2.6
CF1-3	2.7	2.7	2.6	2.6	2.9
CF1-4	2.5	2.6	2.6	2.1	2.7
CF1-5	2.2	2.7	2.2	2.5	2.5
CF1-6	2.4	2.6	2.8	2.6	2.8
CF1-7	2.1	2.3	2.7	2.3	2.4
CF1-8	2.4	2.9	2.5	2.6	2.8
CF1-9	2.3	2.4	2.1	2.5	2.6
CF1-10	2.5	2.4	2.6	2.5	2.6
CF1-11	2.6	2.7	2.7	2.7	2.8
CF2-1	2.7	3.1	2.5	2.7	2.7
CF2-2	2.3	2.4	2.8	2.8	2.7
CF2-3	2.8	2.1	1.7	1.7	1.8
CF2-4	2.3	2.3	2.3	3	2.7
CF2-5	2.9	2.9	2.1	2.8	2.6
CF2-6	2.5	2.1	2.6	2.8	2.5
CF2-7	2.1	2.5	2.8	2.6	2.5
CF2-8	1.8	3	2.9	2.7	2.7
CF2-9	2.5	3.1	2.9	3	2.6
CF2-10	2.3	2.9	2.9	3	2.8
CF2-11	2.4	2.4	2.5	2.6	2.7
CF3-1	2.2	2.3	2.4	2.5	3.5
CF3-2	3.4	3.7	3.3	3.3	3.7
CF3-3	2.6	2.8	3	2.8	3
CF3-4	2.5	2.4	2.7	2.6	3.1
CF3-5	2.2	3.1	3.4	3.4	3.3
CF3-6	2.9	2.9	2.9	3.1	3.2
CF3-7	2.7	2.8	3.5	3.1	3.5
CF3-8	2.7	3.9	3.5	3.6	3.4
CF3-9	2.8	2.8	2.8	2.9	3
CF3-10	1.7	2.8	2.5	2.8	2.7
CF3-11	2.8	2.9	3.5	3.3	3.7
CF3-12	1.6	2.9	3.1	3.1	2.8
CF4-1	2.5	2.6	2.7	2.7	2.8
CF4-2	2.1	2.8	2.8	2.8	3.1
CF4-3	2.9	2.9	3.1	3.3	3.1
CF4-4	2.9	2.9	3.1	3.1	3.7
CF4-5	2.9	3.2	2.8	3.2	3.3
CF4-6	2	3.3	3.1	3.4	3.6
CF4-7	0.23	2.4	2.7	3.6	3.1
CF4-8	2.1	2.4	2.5	2.4	2.8
CF4-9	2.1	3.6	3.3	3.4	3.4
CF4-10	2.8	3	3.6	3	3.1
CF4-11	2.5	2.7	2.7	3	3.5
CF4-12	2.6	2.7	3	3	3

Table 9 TP

Group	0W	1W	2W	3W	4W
CF1-1	6.8	8.1	8.6	8	7.8
CF1-2	6.5	6.4	6.3	5.8	6.6
CF1-3	11.5	9.9	8.6	7.3	7.5
CF1-4	8.1	8.4	6.1	5.9	7.1
CF1-5	7.1	11.4	9.7	8.9	7.3
CF1-6	6.5	8.2	6.9	6.5	6.7
CF1-7	8.9	8.4	8.9	8	7.8
CF1-8	7.1	10.2	9	7.5	7.1
CF1-9	8.2	8.9	7.7	7.7	7.5
CF1-10	8.3	7.3	7.9	8.2	8.1
CF1-11	10.1	9.7	7.9	7.9	7.8
CF2-1	8.8	9.2	7.6	7.9	7.9
CF2-2	7.8	12.4	12	9.9	9
CF2-3	10.2	6.9	6.1	6.3	5.4
CF2-4	5.9	5.8	7.5	7.5	7
CF2-5	10.8	9.1	7.9	7.8	7.2
CF2-6	10	9.4	9.6	8.6	7.6
CF2-7	7.6	7.7	7.9	8.4	8.2
CF2-8	5.6	8.1	7.9	7.8	7.4
CF2-9	7.9	7.8	7.8	8.1	7.2
CF2-10	7.7	8.8	9.1	7.8	7.7
CF2-11	8.5	7.6	7.7	6.7	7.3
CF3-1	7.8	7.9	8.5	8.4	9.2
CF3-2	12.9	12.9	9.6	8.9	8.5
CF3-3	10	10	9	8.1	7.6
CF3-4	9.3	7.4	7.5	7.6	7.4
CF3-5	7	8.4	8.4	7.7	7.3
CF3-6	6.6	7	7	6.8	7
CF3-7	7.8	7	8.1	6.7	7.5
CF3-8	5.9	9	7.8	7.4	7.5
CF3-9	9	9.7	8.9	8.8	8.6
CF3-10	8.7	11.4	10.3	9.2	8
CF3-11	9	10.6	10.5	7.9	7.5
CF3-12	6.6	7.2	7.2	7.3	7
CF4-1	8.9	8.6	8.2	8.2	7.8
CF4-2	6.9	8.9	8.1	7	6.8
CF4-3	9.1	8.1	7.9	7.1	7.7
CF4-4	7.8	9.8	9.6	8.6	7.1
CF4-5	8.3	10	9.8	8.8	8.3
CF4-6	7.2	8.8	7.8	7.8	7.7
CF4-7	9.2	8.8	7.9	8.5	8
CF4-8	11.8	11.2	10.2	9.5	9.2
CF4-9	4.7	6.8	7.2	7.1	7.2
CF4-10	6	6.2	7.2	6.6	6.8
CF4-11	8.7	9	7.9	7.7	7.5
CF4-12	8.7	8.9	8.7	8.1	6.9

Table 10 A:G

Group	0W	1W	2W	3W	4W
CF1-1	0.5	0.6	0.4	0.4	0.6
CF1-2	0.5	0.5	0.6	0.6	0.7
CF1-3	0.3	0.4	0.4	0.5	0.6
CF1-4	0.4	0.5	0.8	0.6	0.6
CF1-5	0.4	0.3	0.3	0.4	0.5
CF1-6	0.6	0.5	0.7	0.7	0.8
CF1-7	0.3	0.4	0.4	0.4	0.5
CF1-8	0.5	0.4	0.4	0.5	0.7
CF1-9	0.4	0.4	0.4	0.5	0.5
CF1-10	0.4	0.5	0.5	0.5	0.6
CF1-11	0.4	0.4	0.5	0.5	0.6
CF2-1	0.4	0.5	0.5	0.5	0.5
CF2-2	0.4	0.2	0.3	0.4	0.4
CF2-3	0.37	0.4	0.4	0.4	0.5
CF2-4	0.6	0.7	0.5	0.7	0.6
CF2-5	0.4	0.5	0.4	0.6	0.6
CF2-6	0.3	0.3	0.4	0.5	0.5
CF2-7	0.4	0.4	0.5	0.4	0.5
CF2-8	0.5	0.6	0.6	0.5	0.6
CF2-9	0.5	0.7	0.6	0.6	0.6
CF2-10	0.4	0.5	0.5	0.6	0.6
CF2-11	0.4	0.5	0.5	0.6	0.6
CF3-1	0.4	0.4	0.4	0.4	0.6
CF3-2	0.4	0.4	0.5	0.6	0.8
CF3-3	0.3	0.4	0.5	0.5	0.7
CF3-4	0.4	0.5	0.6	0.5	0.7
CF3-5	0.5	0.6	0.7	0.8	0.8
CF3-6	0.8	0.7	0.7	0.8	0.8
CF3-7	0.7	0.7	0.8	0.9	0.9
CF3-8	0.8	0.8	0.8	0.9	0.8
CF3-9	0.5	0.4	0.5	0.6	0.7
CF3-10	0.2	0.3	0.3	0.4	0.5
CF3-11	0.5	0.4	0.5	0.9	0.8
CF3-12	0.3	0.7	0.8	0.7	0.7
CF4-1	0.4	0.4	0.5	0.5	0.6
CF4-2	0.4	0.5	0.5	0.7	0.8
CF4-3	0.5	0.6	0.6	0.9	0.7
CF4-4	0.6	0.4	0.5	0.6	1.1
CF4-5	0.5	0.5	0.4	0.6	0.7
CF4-6	0.4	0.6	0.7	0.8	0.9
CF4-7	0.3	0.4	0.5	0.7	0.6
CF4-8	0.2	0.3	0.3	0.3	0.4
CF4-9	0.8	1.1	0.8	0.9	0.9
CF4-10	0.9	0.9	1	0.8	0.9
CF4-11	0.4	0.4	0.5	0.6	0.9
CF4-12	0.4	0.4	0.5	0.6	0.8
